# A common molecular signature of intestinal-type gastric carcinoma indicates processes related to gastric carcinogenesis

**DOI:** 10.18632/oncotarget.23670

**Published:** 2017-12-27

**Authors:** Renata Binato, Everton Cruz Santos, Mariana Boroni, Samia Demachki, Paulo Assumpção, Eliana Abdelhay

**Affiliations:** ^1^ Laboratório de Célula tronco, Centro de Transplante de Medula Óssea (CEMO), Instituto Nacional de Câncer (INCA), Rio de Janeiro, RJ, Brazil; ^2^ Instituto Nacional de Ciência e Tecnologia Para o Controle do Câncer (INCT), Rio de Janeiro, RJ, Brazil; ^3^ Laboratório de Bioinformática e Biologia Computacional, Instituto Nacional de Câncer (INCA), Rio de Janeiro, RJ, Brazil; ^4^ Núcleo de Pesquisas em Oncologia, Universidade Federal do Pará (UFPA), Belém, PA, Brazil

**Keywords:** molecular signature, intestinal-type gastric carcinoma, brazilian molecular profile, common molecular signature worldwide

## Abstract

Gastric carcinoma (GC) is one of the most aggressive cancers and the second leading cause of cancer death in the world. According to the Lauren classification, this adenocarcinoma is divided into two subtypes, intestinal and diffuse, which differ in their clinical, epidemiological and molecular features. Several studies have attempted to delineate the molecular signature of gastric cancer to develop new and non-invasive screening tests that improve diagnosis and lead to new treatment strategies. However, a consensus signature has not yet been identified for each condition. Thus, this work aimed to analyze the gene expression profile of Brazilian intestinal-type GC tissues using microarrays and compare the results to those of non-tumor tissue samples. Moreover, we compared our intestinal-type gastric carcinoma profile with those obtained from populations worldwide to assess their similarity. The results identified a molecular signature for intestinal-type GC and revealed that 38 genes differentially expressed in Brazilian intestinal-type gastric carcinoma samples can successfully distinguish gastric tumors from non-tumor tissue in the global population. These differentially expressed genes participate in biological processes important to cell homeostasis. Furthermore, Kaplan-Meier analysis suggested that 7 of these genes could individually be able to predict overall survival in intestinal-type gastric cancer patients.

## INTRODUCTION

Gastric cancer, one of the most aggressive cancers and the second leading cause of cancer-related death worldwide, is a multifactorial disease affected by lifestyle, aging, socioeconomic factors, dietary behavior and infection [1–3].

The majority of gastric cancers are associated with infections agents, including *Helicobacter pylori* (present in 65% to 80% of cases) and *Epstein-Barr virus* (present in 6% to 10% of cases) [4–5]. Although the role of *Helicobacter pylori* in the emergence of gastric cancer has already been proposed, the role of *Epstein-Barr virus* is not yet clear because only a small group of patients harbor this infection [2, 6–8].

Most gastric cancers are adenocarcinomas that are divided into two subtypes according to the Lauren classification, intestinal and diffuse [9], which differ in their clinical and epidemiological features. Moreover, most cases are sporadic and occur as a result of acquired genetic abnormalities, such as microsatellite instability, changes in the epigenetic landscape, somatic gene mutation or single nucleotide polymorphisms (SNPs) within key candidate genes [5, 10–13].

Late disease detection due to the nonspecific symptomatology in early stages remains a significant problem in gastric cancer that is associated with poor prognosis and a 5-year survival rate of approximately 20%. Moreover, surgical resection and chemotherapy have a limited value for treating patients in the advanced stages [2, 4, 5, 7]. Therefore, many studies have attempted to elucidate gastric cancer biology to develop new non-invasive screening tests that improve diagnosis and facilitate the development of new treatment strategies.

Many innovative technologies have been used in the past five years to identify changes in cell biology associated with gastric cancer. Several genetic abnormalities, such as aberrant genes, copy number variation, microRNAs and long non-coding RNAs, were identified as possible biomarkers in these studies [14–16]. However, the molecular mechanisms leading to gastric cancer and those responsible for its progression remain poorly understood.

Bessède and co-workers [17] suggested that long-term chronic infection that damages the gastric mucosa and recruits bone marrow mesenchymal cells may cause gastric cancer. However, even this model depends on additional epigenetic and mutational events for carcinogenesis.

In addition to the large number of genomic analyses that identified most known mutations related to gastric cancer [18], several studies have attempted to define the gene expression signature of gastric cancer [19–21]. Although these studies successfully correlated some changes in gene expression to specific conditions and resultant abnormalities in cellular processes, a consensus signature has not yet been clearly identified for the two subtypes of gastric cancer.

The distribution of gastric cancer worldwide is heterogeneous, and much of the information in the literature does not apply across all populations. In fact, almost all studies were conducted on populations from Asia or Central America. Therefore, we attempted to broaden the applicability of current findings by analyzing microarrays and comparing the Brazilian intestinal-type gastric carcinoma profile to that of other populations. To address this hypothesis, we used chip arrays to compare the gene expression profiles of tumor samples from Brazilian patients with intestinal-type gastric carcinoma with those of non-tumor tissue from the same patient (control). Specifically, our study identified a molecular signature for Brazilian intestinal-type gastric carcinoma that distinguishes tumor from non-tumor tissue. Moreover, we compared this profile with the ones obtained from other populations to assess the similarity of our intestinal-type gastric carcinoma profile with the profiles observed in the global population. To this end, an unsupervised analysis compared microarrays from different studies worldwide and this Brazilian molecular signature, which revealed that 38 genes from the Brazilian intestinal-type gastric carcinoma molecular signature successfully distinguished intestinal gastric tumors from non-tumor tissue in patients worldwide. Among then, seven genes could individually predict overall survival in intestinal-type gastric cancer patients.

## RESULTS

### Differential gene expression: fifty-seven genes define a Brazilian intestinal-type gastric carcinoma molecular profile

The Lauren classification of intestinal gastric cancer has been extensively studied over the years. Several works related to microarrays and gene expression profiles have already been described for this disease, but significant differences in incidence exist between continents. Although the incidence of this disease is highest in men in northeast Asia (Japan, Korea and China), its incidence is low in North America, Africa, south Asia and Oceania. South America (including Brazil) and Europe are classified as intermediate incidence regions [22]. Therefore, most studies related to this disease focus on northeast Asia. Because the Brazilian population is extremely heterogeneous, identifying relationships between the Lauren classification and gene expression patterns as well as the similarity of these patterns to those in other studies worldwide is challenging.

To identify a global gene expression pattern using tumor tissue from patients with intestinal-type gastric carcinoma and compare this pattern with that of non-tumor control tissue, we performed a comparative transcriptome analysis using an expression chip array assay using a pool of 2 samples in each array.

**Figure 1:**
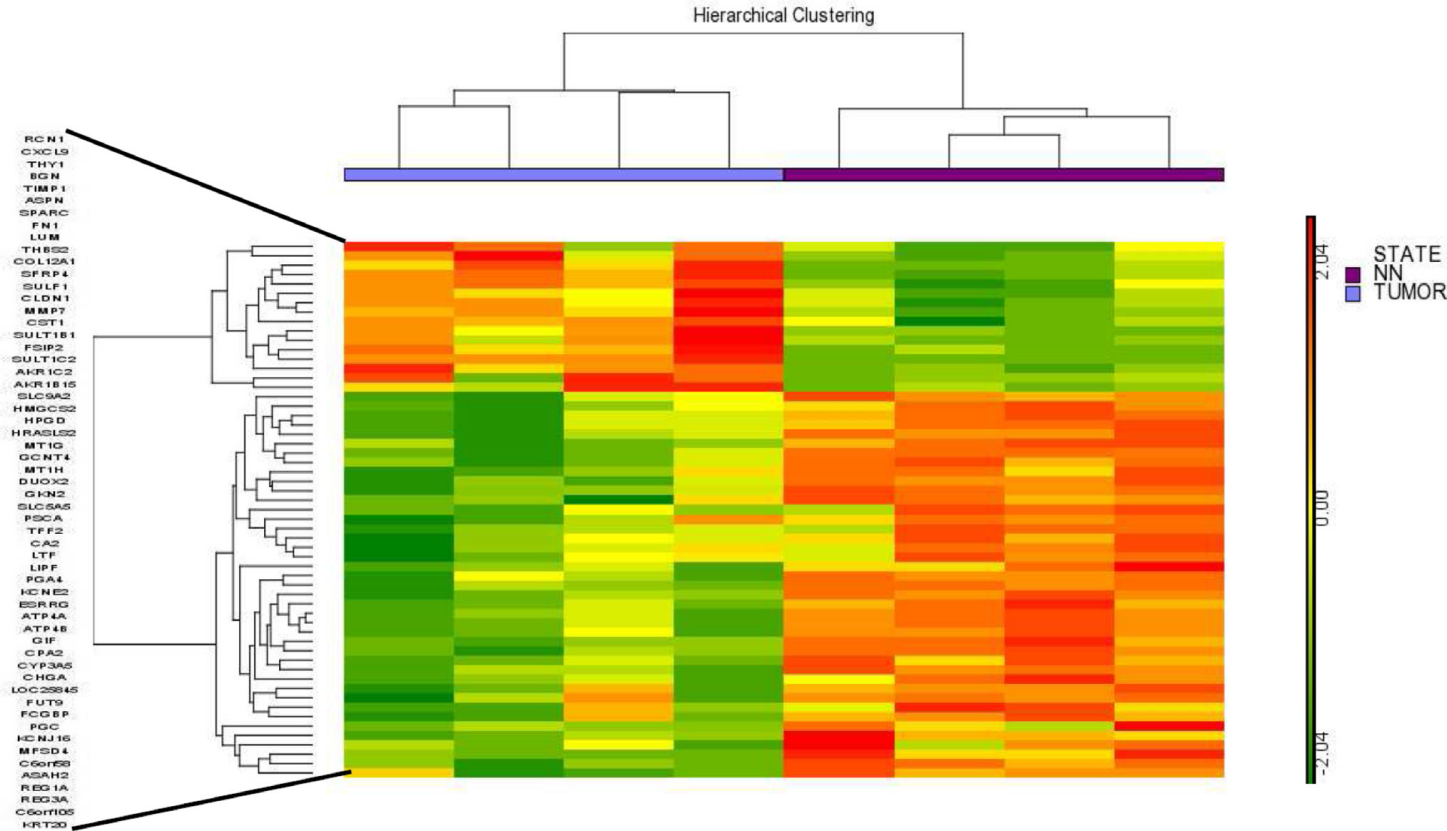
Hierarchical clustering of the 57 differentially expressed genes identified by the chip array assay The results showed a common molecular signature for tumor tissues from intestinal gastric cancer compared to non-tumor control tissues. NN- Non-tumor.

In this assay, 8 samples from patients with different stages of intestinal-type gastric carcinoma were used and compared with the non-tumor control tissue. In total, we have 16 samples, 8 from the tumor region that were divided into 4 arrays containing a pool of two samples in each array and 8 samples from the non-tumor region from the same patients, also divided into 4 arrays containing a pool of two samples in each array. Using a ≥ 5-fold change as the cut-off to define overexpression or downregulation, fifty-seven genes were found to be differentially expressed in all tumor tissue chip array assays. The hierarchical clustering of these differentially expressed genes shown in Figure 1 suggests that a common molecular signature exists for all intestinal-type gastric carcinoma tumors compared to non-tumor control tissues. Interestingly, 16 of these 57 genes were overexpressed in tumor tissues, whereas 41 of these genes were downregulated, indicating a global decrease in gene expression in intestinal gastric cancer tumor tissues (Supplementary Table 1).

### RT-qPCR assay confirmed the Brazilian molecular signature of intestinal-type gastric carcinoma

To confirm the obtained chip array results, quantitative PCR (RT-qPCR) was performed for selected overexpressed (*MMP7*, *SPARC* and *TIMP1*) and downregulated (*CHGA*, *KRT20*, *GIF, AKR1C2* and *PGA4*) genes by comparing tumor and non-tumor tissues in a larger subset of Brazilian patients (*n =* 17). These genes were selected because they had all been previously related to gastric cancer or other cancers.

**Figure 2:**
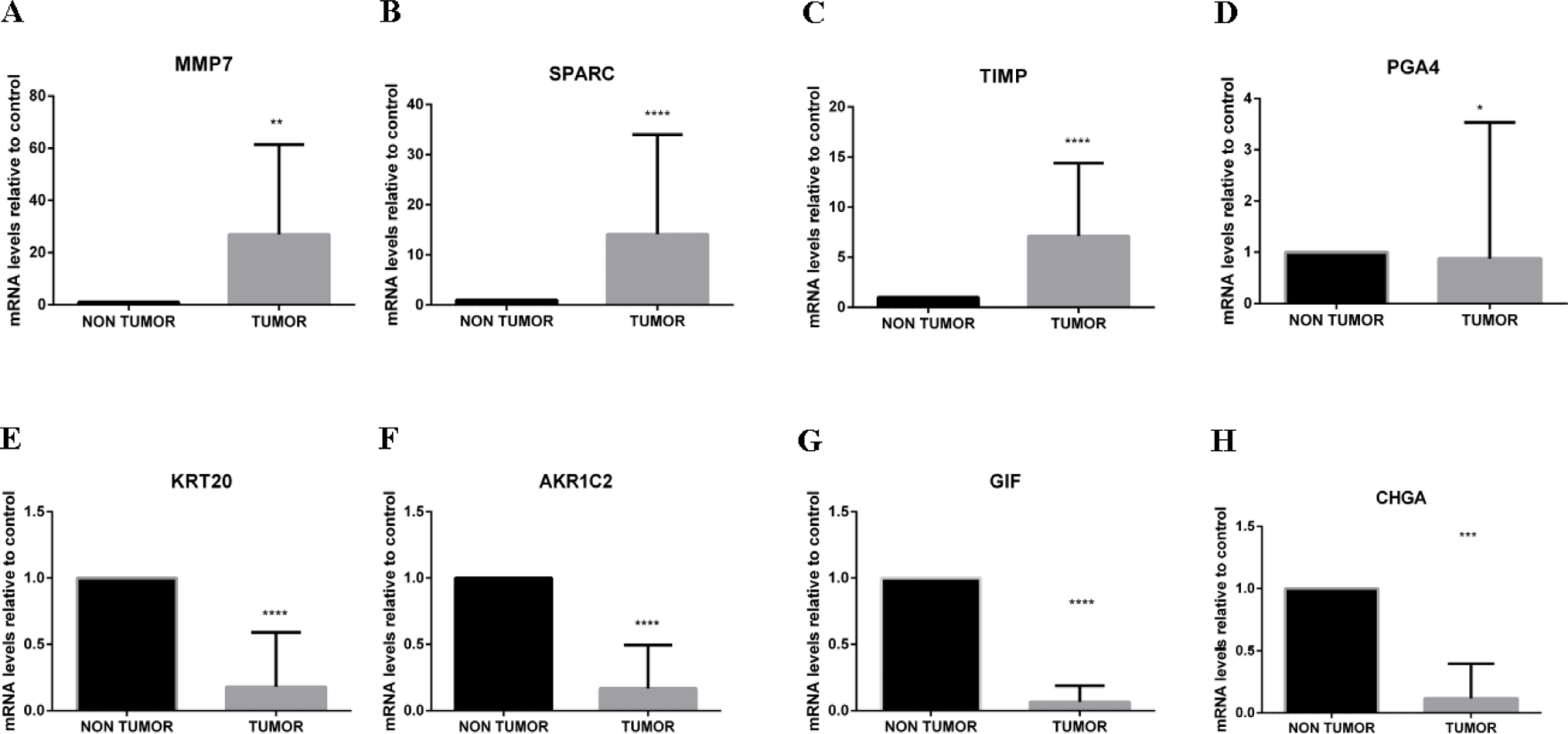
RT-qPCR to validate the chip array assay results To confirm the obtained chip array results, RT-qPCR was used to analyze selected differentially expressed genes using a larger number of Brazilian patient samples to determine changes in mRNA expression levels after normalization to *Actin* and *GAPDH*. RT-qPCR analyses of *MMP7* (**A**), *SPARC* (**B**) and *TIMP1* (**C**) (overexpressed in patients with intestinal gastric cancer) and *PGA4* (**D**), *KRT20* (**E**), *AKR1C2* (**F**), *GIF* (**G**) and *CHGA* (**H**) (downregulated in patients with intestinal gastric cancer) confirmed the chip array assay results and the common molecular signature that was able to discriminate all tumor tissues from all intestinal-type gastric carcinoma patients from non-tumor control tissue. ^*^p < 0.05; ^**^p < 0.01.

The RT-qPCR results presented in Figure 2 confirmed the obtained chip array assay results.

### An unsupervised analysis revealed a common molecular signature for intestinal gastric cancer worldwide

**Figure 3:**
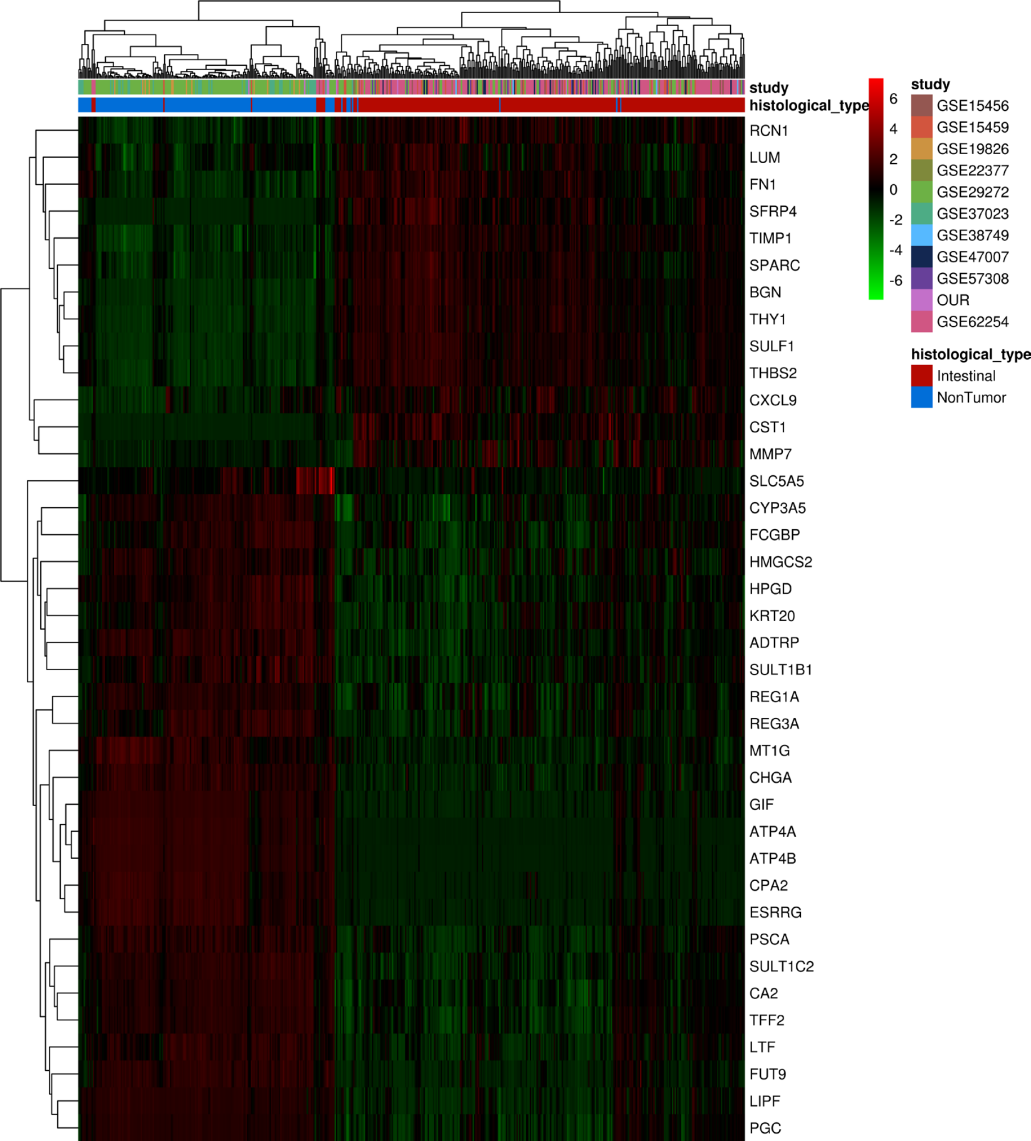
Unsupervised analysis of differentially expressed genes found in Brazilian patients with intestinal-type gastric carcinoma in different populations samples Hierarchical clustering of samples using 38 genes differentially expressed between non-tumor and tumor samples from different studies. Each row represents a gene, and each column represents a sample. The expression level of each gene in a single sample is relative to its median abundance across all samples and is depicted according to a color scale shown at the right. Red and green indicate expression levels above and below the median, respectively. The magnitude of deviation from the median is represented by the color saturation. Dendrograms of samples (above matrix) and genes (to the left of matrix) represent overall similarities in gene expression profiles. For samples, blue boxes represent non-tumor tissue (*n =* 190), and red boxes represent cancerous tissue (*n =* 312). Colored boxes represent datasets from different studies showed in Table 1.

To assess the ability of this molecular signature identified in Brazilian patients with intestinal-type gastric carcinoma to discriminate non-tumor and tumor tissues in samples from intestinal-type gastric carcinoma patients of other nationalities, we performed an unsupervised analysis of 190 non-tumors and 312 tumor samples from different studies representing several countries (Table 1). After the integration of all expression data, only 38 of the 57 differentially expressed genes identified in our dataset were common to all different platforms and could be used in this analysis. An unsupervised, hierarchical clustering of samples based on the expression of these 38 selected genes (Figure 3) successfully distinguished tumor and non-tumor samples. Based on similarities in the expression of this gene panel, the 502 samples separated into two large clusters that extensively differed in terms of disease status (tumor or non-tumor). A small set of tumor samples produced a separate cluster due to the upregulation of most selected genes, suggesting that the tumors can be divided into two types based on this set of 38 significant genes.

**Table 1:** Microarray data from other studies

Study-GEO acession	Microarray-platform	Nationality	Histologycal type
GSE15456	Affymetrix Human Genome U133A Array	United Kingdom	Intestinal
GSE15459	Affymetrix Human Genome U133 Plus 2.0 Array	Singapore	Intestinal
GSE19826	Affymetrix Human Genome U133 Plus 2.0 Array	China	Non Tumor
GSE22377	Affymetrix Human Genome U133 Plus 2.0 Array	Germany	Intestinal
GSE29272	Affymetrix Human Genome U133A Array	China	Intestinal/Non tumor
GSE37023	Affymetrix Human Genome U133A Array	Several Cohorts	NonbTumor
GSE38749	Affymetrix Human Genome U133 Plus 2.0 Array	Brazil	Intestinal
GSE47007	Affymetrix Human Genome U95 Version 2 Array	Japan	Intestinal
GSE57308	Affymetrix Human Genome U133 Plus 2.0 Array	China	Intestinal
GSE62254	Affymetrix Human Genome U133 Plus 2.0	Asian Cancer Research Group cohort	Intestinal

Overall, the results confirmed that this molecular signature can distinguish intestinal-type gastric carcinoma tissue from non-tumor tissue and suggested a common molecular signature for intestinal-type gastric carcinoma, independent of geographic origin of the patient.

### Pathways and processes related to the 38 differentially expressed genes

An *in silico* analysis of the 38 genes defined as the common molecular signature was conducted using the Metacore™ software (GeneGO Inc., Encinitas, CA). This tool categorized the input genes to produce representative pathway maps. As shown in Table 2, the most representative processes that the 38 common differentially expressed genes participated in, were related to matrix alterations, adhesion, gastric mucosa modification, and inflammation. Some overlapping genes, e.g., *TIMP1*, *MMP7* and *FN1*, appeared in two or more of these processes and may be involved in cross-talk between these pathways. The upregulated genes were primarily involved in extracellular matrix remodeling, whereas the downregulated genes were involved in pathways associated with the differentiation and normal function of the gastric mucosa in tumor tissues.

**Table 2:** Processes related to the 38 common genes differentially expressed in intestinal-type gastric carcinoma

Functional Enrichment Analysis^a^	Gene^b^	
	UP	DOWN
Extracellular Matrix Remodeling	TIMP1, MMP-7, FN1, SPARC, LUM	
Gastrin in differentiation of the gastric mucosa		REG1A, CHGA, TFF2
Stimulation of gastric acid secretion		ATP4A, ATP4B, CHGA
Cell adhesion_Cell-matrix interactions	LUM, TIMP1, MMP-7, FN1, BGN	REG3A
Inflamation	TIMP1, CXCL9, FN1	REG3A

^**a**^Enrichment analysis was performed using Metacore™.

^**b**^Gene symbols from 38 genes found in our unsupervised analysis which were identified to be significantly up- or down-regulated in pathway maps.

### The prognostic value of the genes from molecular signature

**Figure 4:**
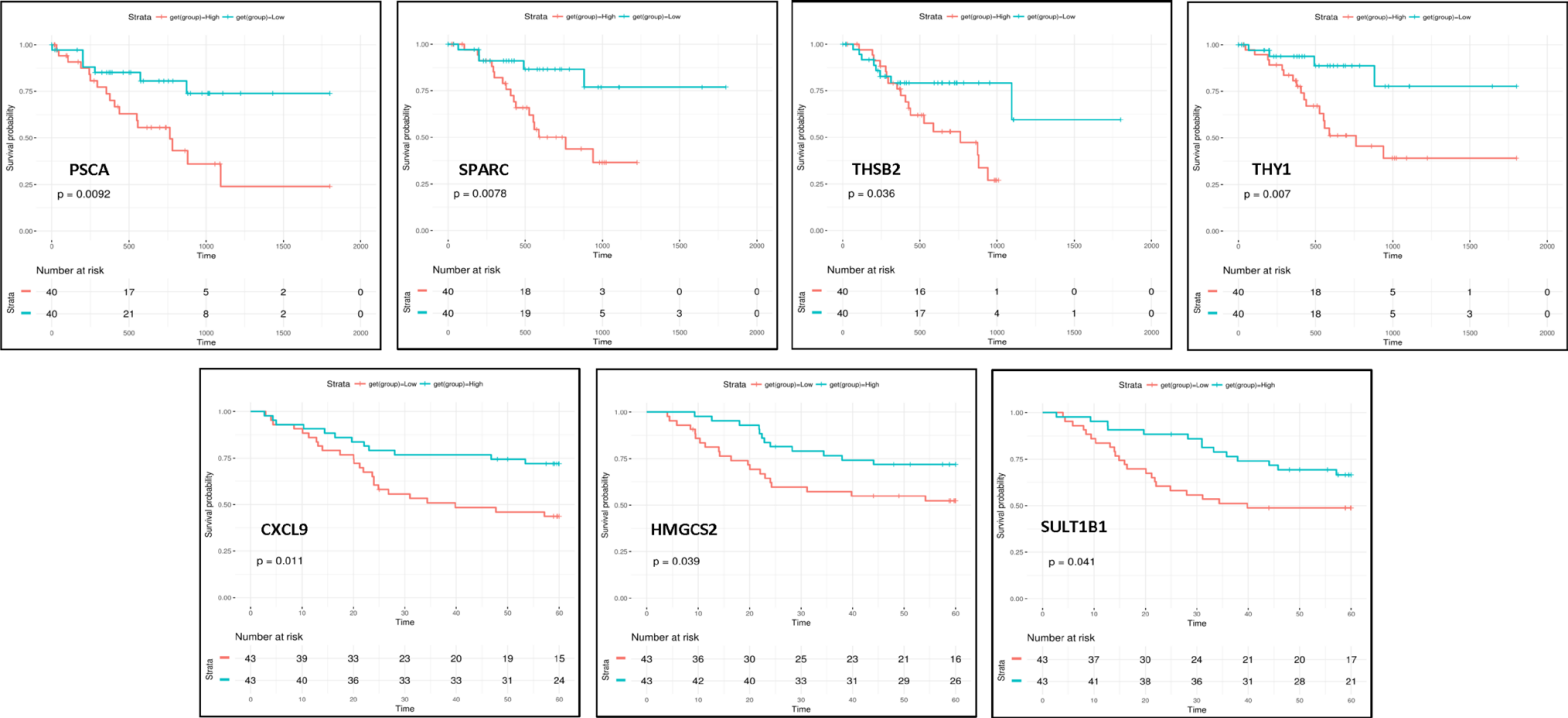
Overall survival of patients stratified according to gene expression Kaplan-Meier analyses showed that patients with high levels of *PSCA*, *SPARC, THBS2* and *THY1* genes had a significantly poor overall survival while high levels of *CXCL9, HMGCS2* and *SULT1B1* genes has a protective effect (*p* < 0.05).

In order to analyze the impact of high expression of the differentially expressed genes found in intestinal-type gastric cancer on overall patient survival we have performed Kaplan-Meier analysis on two validation cohorts of intestinal-type patients that provided overall survival information, one from Microarray data used in our unsupervised analysis and the other one from RNA-seq data from TCGA Stomach adenocarcinoma (TCGA-STAD) dataset [27]. Kaplan-Meier analyses demonstrated that patients with tumors expressing high levels of *PSCA*(*HR*, 3.05; 95% *CI*, 1.26–7.37), *SPARC* (*HR*, 3.56; 95% *CI*, 1.31–9.66)*, THBS2* (*HR*, 2.47; 95% *CI*, 1.03–5.927) and *THY1* (*HR*, 3.40; 95% *CI*, 1.34–11.89) genes had a significantly poor overall survival while high levels of *CXCL9* (*HR*, 0.42; 95% *CI*, 0.21–0.83)*, HMGCS2* (*HR*, 0.48; 95% *CI*, 0.23–0.98)*, SULT1B1* (*HR*, 0.50; 95% *CI*, 0.26–1.00) genes has a protective effect (*p* < 0.05 by the log-rank test) (Figure 4). Altogether these results suggested that these genes could be intestinal-type gastric cancer survival predictors.

## DISCUSSION

Many factors synergistically contribute to cancer development, such as infection, environment and heredity. Although the diagnostic capabilities and therapeutic methods for gastric cancer have improved, the prognosis of patients with gastric cancer remains poor, especially in the advanced stages.

Several groups have used genome and transcriptome profiling to identify genes that could be related to gastric cancer. However, the majority of these studies use samples from populations in which the disease incidence is highest, and few studies have examined populations in which the incidence of this disease is lower [18–21, 23, 24].

In this study, we compared the gene expression profiles of tumor tissue from Brazilian patients with intestinal-type gastric carcinoma and their corresponding non-tumor control tissue using a transcriptome analysis. The molecular profiles of these samples revealed that 57 genes that were differentially expressed compared with non-tumor tissue could differentiate intestinal tumor tissue from non-tumor tissue, suggesting that these genes constituted an intestinal-type gastric carcinoma molecular signature. A RT-qPCR analysis confirmed that this molecular signature can distinguish intestinal tumor tissue from non-tumor control tissue. Thus, this molecular signature may serve as an important molecular marker to identify patients with intestinal gastric cancer in Brazil.

Because the Brazilian population is extremely heterogeneous and the incidence of gastric cancer in our population is intermediate [22], we assessed the similarity of this expression profile to that of other populations worldwide. To this end, we performed an unsupervised analysis using the molecular signature found from Brazilian intestinal gastric cancer and verified the ability of this signature to discriminate non-tumor and tumor samples from other nationalities. Our results show that the 38 genes identified in the Brazilian population are sufficient to discriminate tumor and non-tumor region in patients with intestinal gastric cancers, irrespective of region.

An *in silico* analysis of the 38 differentially expressed genes defined as the common signature of intestinal gastric cancer showed important processes that may be involved in the development or progression of gastric cancer, including extracellular matrix (ECM) remodeling and alterations in cell adhesion, gastric mucosa modification, gastric acid secretion and inflammation.

Pathways that affect the ECM also interact with cell adhesion molecules. This balance between cell adhesion and extracellular molecules is essential for normal cell survival, and imbalance among these pathways results in the detachment of cells from the extracellular matrix and consequently promotes metastasis [25]. Changes in the ECM and cell adhesion processes have been identified in several cancers, suggesting that it plays an essential role in cancer biology. We herein identified a large number of genes associated with the ECM and cell adhesion to be differentially expressed in intestinal gastric cancer, including *TIMP1*, *MMP7*, *FN1, SPARC, LUM* and *BGN*, which were upregulated.

*MMP7* is a matrix metalloprotease gene that is involved in the degradation of all components of basement membranes under physiological conditions. Under pathological conditions, *MMP7* overexpression has been associated with cancer-cell invasion and metastasis, and *MMP7* regulates cancer-associated processes, such as the inhibition of apoptosis, the degradation of cell-cell contact and cellular proliferation [26–28]. In gastric cancer, *MMP7* was previously identified to be overexpressed, and Koskensalo and co-workers suggested that this gene may be an independent prognostic marker [29]. Moreover, the *SPARC* gene encodes a matrix-associated protein that is required for the calcification of collagen in bone but is also involved in extracellular matrix synthesis and changes in cell shape. Its gene product has been correlated with metastasis based on changes in cell shape, which can promote tumor cell invasion [30]. Specifically, the expression of *SPARC* is higher in advanced gastric cancer compared to the early stages, and high *SPARC* expression significantly correlated with lymph node metastasis, lymphatic invasion and perineural invasion [31]. Furthermore, *TIMP1* is a metallopeptidase inhibitor 1 gene that is involved in the control of the proteolytic activities of MMPs during the degradation of the extracellular matrix. *TIMP1* can also induce cell proliferation and has an anti-apoptotic effect, and its overexpression has been associated with a poor prognosis in several types of cancer [32–34]. *TIMP1* has been reported to be overexpressed in gastric cancer cells and in the inflammatory cells of the stromal element of the tumor, and high levels of this protein are associated with poor outcome [35, 36]. The *FN1* gene encodes fibronectin, a ubiquitous ECM protein related to many important normal biologic processes, such as cell adhesion and migration. In several cancers, *FN1* is a key mediator of disease progression and metastasis [37–40]. In gastric cancer tissue, *FN1* expression was found to be upregulated and related to invasion and migration [41, 42]. Moreover, the lumican gene (*LUM*) is also a component of the ECM that participates in important regulatory processes, such as cell proliferation, migration and adhesion. Additionally, *LUM* has been associated with the aggressiveness of lung adenocarcinoma and squamous cell carcinoma and was found to be overexpressed in gastric cancer [43, 44]. Biglycan (*BGN*) is expressed in the ECM, and its upregulation was associated with several types of cancer, including colon tumor, pancreatic cancer and gastric cancer [45–48]. GC cells secrete BGN into the tumor stroma and promote GC progression [48, 49]. This protein may also regulate inflammation and innate immunity.

Other processes that seem to be important in intestinal gastric cancer are gastric acid secretion and the differentiation of gastric mucosa. We identified *REG1A, CHGA, TFF2, ATP4A* and *ATP4B* to be downregulated in intestinal gastric cancer, and Rajkumar and co-workers found *ATP4A* and *ATP4B* to be downregulated in gastric tumor tissues compared to normal tissues [44]. ATPases are the most critical component of the ion transport system in parietal cells, which mediate acid secretion in the stomach and the inhibition of ATPase activity cause epithelial cell proliferation and suppress their differentiation [50]. *TFF* genes play a regulatory role in the mammalian digestive system, specifically in mucosal protection and epithelial cell reconstruction, tumor suppression or promotion, signal transduction and the regulation of proliferation and apoptosis. *TFF2* expression is high in the normal gastric mucosa, and several studies have shown that *TFF2* expression is downregulated in gastric cancer compared with normal tissue and that this downregulation may be associated with promoter hypermethylation [51]. The *REG1A* gene encodes a protein that is secreted by the exocrine pancreas [52] and is expressed in the normal colorectal mucosa and tumors, such colorectal cancer, pancreatic ductal adenocarcinoma [53–56]. Zhang and co-workers used an RNA-seq approach to identify that *REG1A* was downregulated in gastric carcinoma [23]. Chromogranin A (*CHGA*) belongs to the granins (acidic glycoproteins) family, which is related to the family of neuroendocrine secretory proteins, and it is crucial for the exocytosis of secretory vesicles in neuroendocrine cells, including the gastrointestinal endocrine system [57, 58]. Signet ring cells (SRC) were found to be derived from neuroendocrine cells, indicating that SRC-gastric carcinomas may be of neuroendocrine origin [59]. *CHGA* expression correlates with better prognosis in SRC-gastric carcinoma [60]. However, the expression of this gene in intestinal-type GC has not yet been described.

Inflammation is also dysregulated in intestinal gastric cancer. The relationship between inflammation and cancer was first discovered in 1863 by Rudolf Virchow, who suggested that cancer may originate at sites of inflammation. Chronic inflammation may increase the risk of developing cancer; for instance, esophagitis or gastritis may lead to the development of esophageal or gastric cancer, respectively [61]. In the common gene signature identified in this study, genes related to inflammation were both up- (*CXCL9* and *FN1*) and downregulated (*REG3A*) in our analysis. The *REG3A* gene has been reported to be downregulated in gastric cancers and may be involved in cell adhesion and protection from oxidative stress-induced apoptosis. *REG3A* has also been reported to bind fibronectin (*FN1*) and is implicated in cell-cell interaction, differentiation and metastasis [62]. *CXCL9* is a C-X-C Motif chemokine ligand that encodes a protein thought to be involved in T cell trafficking [61].

Interestingly, a common expression pattern of 38 genes was consistently associated with intestinal-type gastric carcinoma worldwide, irrespective of the incidence of the disease or heterogeneity of the population. This molecular signature includes genes that participate in processes important to cell homeostasis. This common signature may be useful as a molecular profile of intestinal gastric cancers and warrant exploration since our data indicate a reproducible worldwide framework for this histological type.

Moreover, among these 38 genes, *CXCL9, HMGCS2, SULT1B1, PSCA*, *SPARC*, *THBS2* and *THY1* could predict overall survival. This new gene panel may help guide investigations of new targets to develop novel therapies and customize treatment to improve the overall survival of patients with intestinal gastric cancer.

## MATERIALS AND METHODS

### Patient samples

All tumor tissues and non-tumor control tissues were obtained from patients diagnosed with gastric adenocarcinoma intestinal type by the Lauren classification at the Hospital João de Barros Barreto, Universidade Federal do Pará (Belém, PA, Brazil). The 44 samples from 22 patients obtained were characterized as shown in Table 3. These patients were stratified into two cohorts: chip array cohort (*n =* 8) and RT-qPCR cohort (*n =* 17). All samples were obtained in accordance with the guidelines of the local Ethics Committee and the Helsinki Declaration. The procedures were previously approved by the institutional review board, and all participants signed informed consent forms. This study was approved by the National Ethics Committee (Conselho Nacional de Ética em Pesquisa–CONEP) and the local institutional committee.

**Table 3:** List of Brazilian patients with intestinal-type gastric carcinoma that participated in this study

Sample laboratory code	TNM classification	Chiparray cohort	RT-qPCR confirmation cohor
P1-T	pT3N3	X	
P1-NT	-	X	
P2-T	pT3N3	X	
P2-NT	-	X	
P3-T	pT3N2	X	
P3-NT	-	X	
P4-T	pT2N2	X	
P4-NT	-	X	
P5-T	pT4aN2	X	
P5-NT	-	X	
P6-T	pT4aN2	X	X
P6-NT	-	X	X
P7-T	pT3N3a	X	X
P7-NT	-	X	X
P8-T	pT3N3b	X	X
P8-NT	-	X	X
P9-T	pT4aN3bM1		X
P9-NT	-		X
P10-T	pT3N2		X
P10-NT	-		X
P11-T	pT3N2		X
P11-NT	-		X
P12-T	pT3N1		X
P12-NT	-		X
P13-T	pT3N2		X
P13-NT	-		X
P14-T	pT4bN3a		X
P14-NT	-		X
P15-T	pT4aN3aM1		X
P15-NT	-		X
P16-T	pT4aN3aM1		X
P16-NT	-		X
P17-T	pT4bN2		X
P17-NT	-		X
P18-T	pT4bN3bM1		X
P18-NT	-		X
P19-T	pT3N2		X
P19-NT	-		X
P20-T	pT4N3a		X
P20-NT	-		X
P21-T	pT3N3a		X
P21-NT	-		X
P22-T	pT4bN3b		X

T- TUMOR; NT- NON-TUMOR.

### Expression chip array data analysis

An RNeasy Mini kit (Qiagen, CA, USA) was used to obtain total RNA from intestinal-type gastric carcinoma and non-tumor control tissues according to the manufacturer’s instructions. One hundred nanograms (100 ng) of total RNA were used to synthesize biotinylated cRNA using a GeneChip Whole Transcription (WT) Sense Target Labeling Assay Kit (Affymetrix, CA, USA). The biotinylated cRNA was then hybridized to GeneChip Human Exon 1.0 ST Arrays (Affymetrix, CA, USA), washed and stained according to the manufacturer’s protocols. The GeneChip arrays were scanned using a GeneChip^®^ Scanner 3000. The Affymetrix Expression Console software version 1.0 was used to create summarized expression values (CHP-files), and the robust multichip analysis (RMA) algorithm was applied. The data were analyzed using the Partek^®^ software **(**http://www.partek.com) [63], and a ≥5-fold change in expression was defined as differential overexpression or downregulation. The pathway analysis and related processes were obtained using the MetaCore™ software (http://thomsonreuters.com/metacore/).

### Quantitative PCR (RT-qPCR)

RT-qPCR analyses were performed using 2 μg of mRNA treated with amplification-grade DNase I (Invitrogen, CA, USA) and reverse transcribed with Superscript III Reverse transcriptase^®^ (Invitrogen, CA, USA). Each reaction was performed with 5 μL of SYBR Green PCR Master Mix^®^ (Applied Biosystems, CA, USA), 2.5 μL of cDNA (10 ng of cDNA) and 2 μM of each primer. The mRNA levels were quantified using the Rotor-Gene 6000 Series software (Corbett, Australia). The reactions were performed in a Rotor-Gene 6000 thermocycler (Corbett, Australia) using the following program: 95°C for 5 min, followed by 45 cycles at 95°C for 15 s with a final extension at 62°C for 40 s. A dissociation curve analysis was used to demonstrate that the amplification efficiency of a specific PCR products for all primers used in this study was equal and that products were specific. The fold-change in expression was calculated using the DDCt method according to Livak and Schmittgen [64]. The expression levels were estimated in triplicate, and *Actin* and *GAPDH* were used to normalize gene expression. The following primers were used: *TIMP1* Fw (5′-CATC CTGTTGTTGCTGTGGCTGA-3′) and Rev (5′-GGTGG TCTGGTTGACTTCTGGTGT-3′); *PGA4* Fw (5′-GCCCA GGATTTCACCGTCGTCTT-3′) and Rev (5′-ACTGTCT CGCTGGTGGACTGGTA-3′); *GIF* Fw (5′- ATCTAAC CATTGGGCAGCTCGGC-3′) and Rev (5′-GGCCCATAG AAGGCTGATGCTTCAG-3′); *KRT20* Fw (5′-AGCAGT GGTACGAAACCAACGC-3′) and Rev (5′- CAGGACAC ACCGAGCATTTTGCA-3′); *CHGA* Fw (5′-GCTCCCT GTGAACAGCCCTATGAA-3′) and Rev (5′-GGCTTGGA AAGTGTGTCGGAGATG-3′); *MMP7* Fw (5′-TGCAGA AGCCCAGATGTGGAGTG-3′) and Rev (5′-CGATCCT GTAGGTGACCACTTTGG-3′); *SPARC* Fw (5′-TGCCTG ATGAGACA GAGGTGGT-3′) and Rev (5′-CGGTTT CCTCTGCACCATCA TCAA-3′); *AKR1C2* Fw (5′-AAGCTCTAGAGGCCGTCAAATTGG-3′) and Rev (5′-CTC TGGTCGATGGGAATTGCTCC-3′) *GAPDH* Fw (5′-GT CAACGGATTTGGTC GTATTG-3′) and Rev (5′-TGGAA GATGGTGATGGGATTT-3′), *Actin* Fw (5′-TTCCTTC CTGGGCATGGAGTC-3′) and Rev (5′-AGACAGC ′). The results were compared using the Mann–Whitney test. The GraphPad Prism™ software (GraphPad Software Inc., CA, USA) was used for the statistical analysis and to prepare graphs.

### Unsupervised analysis

Cell intensity (CEL) files storing probe-level intensity data were downloaded from NCBI’s Gene Expression Omnibus (GEO); accession numbers are described in Table 2. The *simpleAffy* Bioconductor R package was used to preprocess all raw data files. The extraction of probe level data, background correction, normalization using the robust multi-array average (RMA) algorithm, and the mapping of probes to genes were performed for each individual experiment to summarize gene-levels of expression. The datasets were then merged to obtain complete expression data. Non-biological experimental variation or batch effects were adjusted using a parametric empirical Bayes framework using the ComBat function implemented on the sva Bioconductor R package [65].

In the two-dimensional cluster analysis, gene clustering and sample clustering were independently performed using an unsupervised hierarchical clustering algorithm. For gene clustering, pairwise similarity metrics among genes were calculated based on expression ratio measurements across all samples (average linkage clustering using Pearson’s correlation as similarity metric). Similarly, for sample clustering, pairwise similarity measures among samples were calculated using the Euclidean distance based on expression ratio measurements across all significant genes.

### TCGA data

Public available RNA-Seq and clinical data from 158 intestinal-type gastric cancer and 15 normal tissues samples from The Cancer Genome Atlas (TCGA) project was downloaded from the NCI’s Genomic Data Commons (GDC) [66] using CGAbiolinks Bioconductor R package [67]. The downloaded files correspond to the clinical data and the HTSeq - counts (gene expression quantification - transcriptome profiling) from the “TCGA Stomach adenocarcinoma (TCGA-STAD) dataset [68]. HTSeq counts were normalized using DESeq2 [69].

### Survival analysis

For survival analysis of the 38 individual marker genes, tumor *samples were stratified into* quartiles *according* to the expression of each marker: the lower quartile was named Low expression group and the upper, High expression group. The survival curves were computed using the method of Kaplan-Meier and Cox proportional hazards models (survival and survminer R package). Statistical significance was determined using the log-rank test.

### Statistical analysis

All experiments were carried out in triplicate, and the data are expressed as the mean ± standard error of the mean. The results were compared using an unpaired Mann–Whitney test, and a *p*-value <0.05 was considered significant (^*^p < 0.05, ^**^p < 0.01). The GraphPad Prism™ software (GraphPad Software Inc., CA, USA) was used for statistical analyses and to generate graphs.

## SUPPLEMENTARY MATERIALS TABLE




