# Supplementary material for: A common molecular signature of intestinal-type gastric carcinoma indicates processes related to gastric carcinogenesis
Source: Oncotarget. 2017 Dec 27;9(7):7359–71. doi: 10.18632/oncotarget.23670 (PMC5800908; doi:10.18632/oncotarget.23670)
Supplement: Supplementary file 1 [file oncotarget-09-7359-s001.pdf]

A common molecular signature of intestinal-type gastric carcinoma indicates processes related to gastric carcinogenesis

SUPPLEMENTARY MATERIALS

Supplementary Table 1: List of the 57 differentially expressed genes identified by chip array assay. See Supplementary_Table_1
