# Supplementary material for: A common molecular signature of intestinal-type gastric carcinoma indicates processes related to gastric carcinogenesis
Source: Oncotarget. 2017 Dec 27;9(7):7359–71. doi: 10.18632/oncotarget.23670 (PMC5800908; doi:10.18632/oncotarget.23670)
Supplement: Supplementary file 2 [file oncotarget-09-7359-s002.doc]

**Supplementary Table 1**: **List of the 57 differentially expressed genes identified by chip array assay**

| **Gene Symbol** | **Gene_assignment** | **RefSeq** | **Fold-Change** | **TUMOR x NON TUMOR** | **Transcript ID** |
| --- | --- | --- | --- | --- | --- |
| AKR1B15 | NM_001080538 // AKR1B15 // aldo-keto reductase family 1, member B15 // 7q33 // 441282 / | NM_001080538 | -19,62 | TUMOR down x NON TUMOR | 3025433 |
| AKR1C2 | NM_205845 // AKR1C2 // aldo-keto reductase family 1, member C2 (dihydrodiol dehydrogena | NM_205845 | -5,13 | TUMOR down x NON TUMOR | 3274758 |
| ASAH2 | NM_019893 // ASAH2 // N-acylsphingosine amidohydrolase (non-lysosomal ceramidase) 2 // | NM_019893 | -6,91 | TUMOR down x NON TUMOR | 3289189 |
| ASPN | NM_017680 // ASPN // asporin // 9q22 // 54829 /// ENST00000375544 // ASPN // asporin // | NM_017680 | 7,62 | TUMOR up x NON TUMOR | 3214845 |
| ATP4A | NM_000704 // ATP4A // ATPase, H+/K+ exchanging, alpha polypeptide // 19q13.1 // 495 /// | NM_000704 | -23,00 | TUMOR down x NON TUMOR | 3859832 |
| ATP4B | NM_000705 // ATP4B // ATPase, H+/K+ exchanging, beta polypeptide // 13q34 // 496 /// EN | NM_000705 | -26,94 | TUMOR down x NON TUMOR | 3526655 |
| BGN | NM_001711 // BGN // biglycan // Xq28 // 633 /// ENST00000331595 // BGN // biglycan // X | NM_001711 | 6,69 | TUMOR up x NON TUMOR | 3995633 |
| C6orf105 | NM_001143948 // C6orf105 // chromosome 6 open reading frame 105 // 6p24.1 // 84830 /// | NM_001143948 | -10,73 | TUMOR down x NON TUMOR | 2941972 |
| C6orf58 | AK303850 // C6orf58 // chromosome 6 open reading frame 58 // 6q22.33 // 352999 /// ENST | AK303850 | -5,55 | TUMOR down x NON TUMOR | 2925013 |
| CA2 | NM_000067 // CA2 // carbonic anhydrase II // 8q22 // 760 /// ENST00000285379 // CA2 // | NM_000067 | -5,51 | TUMOR down x NON TUMOR | 3105600 |
| CHGA | NM_001275 // CHGA // chromogranin A (parathyroid secretory protein 1) // 14q32 // 1113 | NM_001275 | -5,40 | TUMOR down x NON TUMOR | 3549092 |
| CLDN1 | NM_021101 // CLDN1 // claudin 1 // 3q28-q29 // 9076 /// ENST00000295522 // CLDN1 // cla | NM_021101 | 9,75 | TUMOR up x NON TUMOR | 2710599 |
| COL12A1 | NM_004370 // COL12A1 // collagen, type XII, alpha 1 // 6q12-q13 // 1303 /// NM_080645 / | NM_004370 | 5,40 | TUMOR up x NON TUMOR | 2961177 |
| CPA2 | NM_001869 // CPA2 // carboxypeptidase A2 (pancreatic) // 7q32 // 1358 /// BC014571 // C | NM_001869 | -6,90 | TUMOR down x NON TUMOR | 3023835 |
| CST1 | NM_001898 // CST1 // cystatin SN // 20p11.21 // 1469 /// ENST00000304749 // CST1 // cys | NM_001898 | 7,67 | TUMOR up x NON TUMOR | 3901361 |
| CXCL9 | NM_002416 // CXCL9 // chemokine (C-X-C motif) ligand 9 // 4q21 // 4283 /// ENST00000264 | NM_002416 | 5,81 | TUMOR up x NON TUMOR | 2773947 |
| CYP3A5 | NM_000777 // CYP3A5 // cytochrome P450, family 3, subfamily A, polypeptide 5 // 7q21.1 | NM_000777 | -5,35 | TUMOR down x NON TUMOR | 3063406 |
| DUOX2 | NM_014080 // DUOX2 // dual oxidase 2 // 15q15.3 // 50506 /// NM_017434 // DUOX1 // dual | NM_014080 | -6,49 | TUMOR down x NON TUMOR | 3622176 |
| ESRRG | NR_024099 // ESRRG // estrogen-related receptor gamma // 1q41 // 2104 /// NM_206594 // | NR_024099 | -7,03 | TUMOR down x NON TUMOR | 2455933 |
| FCGBP | NM_003890 // FCGBP // Fc fragment of IgG binding protein // 19q13.1 // 8857 /// ENST000 | NM_003890 | -10,76 | TUMOR down x NON TUMOR | 3862188 |
| FN1 | NM_212482 // FN1 // fibronectin 1 // 2q34 // 2335 /// NM_212475 // FN1 // fibronectin 1 | NM_212482 | 6,34 | TUMOR up x NON TUMOR | 2598261 |
| FSIP2 | AK092099 // FSIP2 // fibrous sheath interacting protein 2 // 2q32.1 // 401024 /// BC121 | AK092099 | -6,20 | TUMOR down x NON TUMOR | 2519038 |
| FUT9 | NM_006581 // FUT9 // fucosyltransferase 9 (alpha (1,3) fucosyltransferase) // 6q16 // 1 | NM_006581 | -5,27 | TUMOR down x NON TUMOR | 2917825 |
| GCNT4 | NM_016591 // GCNT4 // glucosaminyl (N-acetyl) transferase 4, core 2 // 5q12 // 51301 // | NM_016591 | -6,31 | TUMOR down x NON TUMOR | 2862841 |
| GIF | NM_005142 // GIF // gastric intrinsic factor (vitamin B synthesis) // 11q13 // 2694 /// | NM_005142 | -45,48 | TUMOR down x NON TUMOR | 3374874 |
| GKN2 | NM_182536 // GKN2 // gastrokine 2 // 2p13.3 // 200504 /// ENST00000328895 // GKN2 // ga | NM_182536 | -8,24 | TUMOR down x NON TUMOR | 2557956 |
| HMGCS2 | NM_005518 // HMGCS2 // 3-hydroxy-3-methylglutaryl-CoA synthase 2 (mitochondrial) // 1p1 | NM_005518 | -5,81 | TUMOR down x NON TUMOR | 2431031 |
| HPGD | NM_000860 // HPGD // hydroxyprostaglandin dehydrogenase 15-(NAD) // 4q34-q35 // 3248 // | NM_000860 | -6,95 | TUMOR down x NON TUMOR | 2794408 |
| HRASLS2 | NM_017878 // HRASLS2 // HRAS-like suppressor 2 // 11q12.3 // 54979 /// ENST00000255695 | NM_017878 | -7,55 | TUMOR down x NON TUMOR | 3376512 |
| KCNE2 | NM_172201 // KCNE2 // potassium voltage-gated channel, Isk-related family, member 2 // | NM_172201 | -23,46 | TUMOR down x NON TUMOR | 3919101 |
| KCNJ16 | NM_170742 // KCNJ16 // potassium inwardly-rectifying channel, subfamily J, member 16 // | NM_170742 | -5,29 | TUMOR down x NON TUMOR | 3733238 |
| KRT20 | NM_019010 // KRT20 // keratin 20 // 17q21.2 // 54474 /// ENST00000167588 // KRT20 // ke | NM_019010 | -11,58 | TUMOR down x NON TUMOR | 3756566 |
| LIPF | NM_004190 // LIPF // lipase, gastric // 10q23.31 // 8513 /// ENST00000238983 // LIPF // | NM_004190 | -44,84 | TUMOR down x NON TUMOR | 3256914 |
| LOC25845 | NR_024158 // LOC25845 // hypothetical LOC25845 // 5p15.33 // 25845 /// AK074086 // EXOC | NR_024158 | -6,11 | TUMOR down x NON TUMOR | 2845351 |
| LTF | NM_002343 // LTF // lactotransferrin // 3p21.31 // 4057 /// ENST00000231751 // LTF // l | NM_002343 | -5,10 | TUMOR down x NON TUMOR | 2672140 |
| LUM | NM_002345 // LUM // lumican // 12q21.3-q22 // 4060 /// ENST00000266718 // LUM // lumica | NM_002345 | 6,95 | TUMOR up x NON TUMOR | 3465248 |
| MFSD4 | NM_181644 // MFSD4 // major facilitator superfamily domain containing 4 // 1q32.1 // 14 | NM_181644 | -5,35 | TUMOR down x NON TUMOR | 2376548 |
| MMP7 | NM_002423 // MMP7 // matrix metallopeptidase 7 (matrilysin, uterine) // 11q21-q22 // 43 | NM_002423 | 7,40 | TUMOR up x NON TUMOR | 3388673 |
| MT1G | NM_005950 // MT1G // metallothionein 1G // 16q13 // 4495 /// BC020757 // MT1G // metall | NM_005950 | -5,83 | TUMOR down x NON TUMOR | 3692999 |
| MT1H | NM_005951 // MT1H // metallothionein 1H // 16q13 // 4496 /// NM_005949 // MT1F // metal | NM_005951 | -5,23 | TUMOR down x NON TUMOR | 3662130 |
| PGA4 | NM_001079808 // PGA4 // pepsinogen 4, group I (pepsinogen A) // 11q12.2 // 643847 /// N | NM_001079808 | -50,00 | TUMOR down x NON TUMOR | 3332780 |
| PGC | NM_002630 // PGC // progastricsin (pepsinogen C) // 6p21.3-p21.1 // 5225 /// NM_0011664 | NM_002630 | -13,47 | TUMOR down x NON TUMOR | 2953751 |
| PSCA | NM_005672 // PSCA // prostate stem cell antigen // 8q24.2 // 8000 /// NR_033343 // PSCA | NM_005672 | -8,66 | TUMOR down x NON TUMOR | 3119200 |
| RCN1 | NM_002901 // RCN1 // reticulocalbin 1, EF-hand calcium binding domain // 11p13 // 5954 | NM_002901 | 5,84 | TUMOR up x NON TUMOR | 3325503 |
| REG1A | NM_002909 // REG1A // regenerating islet-derived 1 alpha // 2p12 // 5967 /// ENST000002 | NM_002909 | -6,69 | TUMOR down x NON TUMOR | 2490324 |
| REG3A | NM_138938 // REG3A // regenerating islet-derived 3 alpha // 2p12 // 5068 /// NM_002580 | NM_138938 | -18,10 | TUMOR down x NON TUMOR | 2561216 |
| SFRP4 | NM_003014 // SFRP4 // secreted frizzled-related protein 4 // 7p14.1 // 6424 /// ENST000 | NM_003014 | 5,33 | TUMOR up x NON TUMOR | 3046444 |
| SLC5A5 | NM_000453 // SLC5A5 // solute carrier family 5 (sodium iodide symporter), member 5 // 1 | NM_000453 | -7,24 | TUMOR down x NON TUMOR | 3824623 |
| SLC9A2 | NM_003048 // SLC9A2 // solute carrier family 9 (sodium/hydrogen exchanger), member 2 // | NM_003048 | -5,26 | TUMOR down x NON TUMOR | 2845362 |
| SPARC | NM_003118 // SPARC // secreted protein, acidic, cysteine-rich (osteonectin) // 5q31.3-q | NM_003118 | 5,33 | TUMOR up x NON TUMOR | 2882098 |
| SULF1 | NM_001128205 // SULF1 // sulfatase 1 // 8q13.2-q13.3 // 23213 /// NM_015170 // SULF1 // | NM_001128205 | 6,17 | TUMOR up x NON TUMOR | 3102372 |
| SULT1B1 | NM_014465 // SULT1B1 // sulfotransferase family, cytosolic, 1B, member 1 // 4q13.3 // 2 | NM_014465 | -6,38 | TUMOR down x NON TUMOR | 2772414 |
| SULT1C2 | NM_001056 // SULT1C2 // sulfotransferase family, cytosolic, 1C, member 2 // 2q11.1-q11. | NM_001056 | -10,06 | TUMOR down x NON TUMOR | 2498911 |
| TFF2 | NM_005423 // TFF2 // trefoil factor 2 // 21q22.3 // 7032 /// ENST00000291526 // TFF2 // | NM_005423 | -9,71 | TUMOR down x NON TUMOR | 3933550 |
| THBS2 | NM_003247 // THBS2 // thrombospondin 2 // 6q27 // 7058 /// ENST00000366787 // THBS2 // | NM_003247 | 5,62 | TUMOR up x NON TUMOR | 2985781 |
| THY1 | NM_006288 // THY1 // Thy-1 cell surface antigen // 11q22.3-q23 // 7070 /// ENST00000284 | NM_006288 | 5,60 | TUMOR up x NON TUMOR | 3394412 |
| TIMP1 | NM_003254 // TIMP1 // TIMP metallopeptidase inhibitor 1 // Xp11.3-p11.23 // 7076 /// EN | NM_003254 | 5,05 | TUMOR up x NON TUMOR | 3976341 |
